# Supplementary figures and images for: The function of a heterozygous p53 mutation in a Li-Fraumeni syndrome patient
Source: PLoS One. 2020 Jun 9;15(6):e0234262. doi: 10.1371/journal.pone.0234262 (PMC7282642; doi:10.1371/journal.pone.0234262)

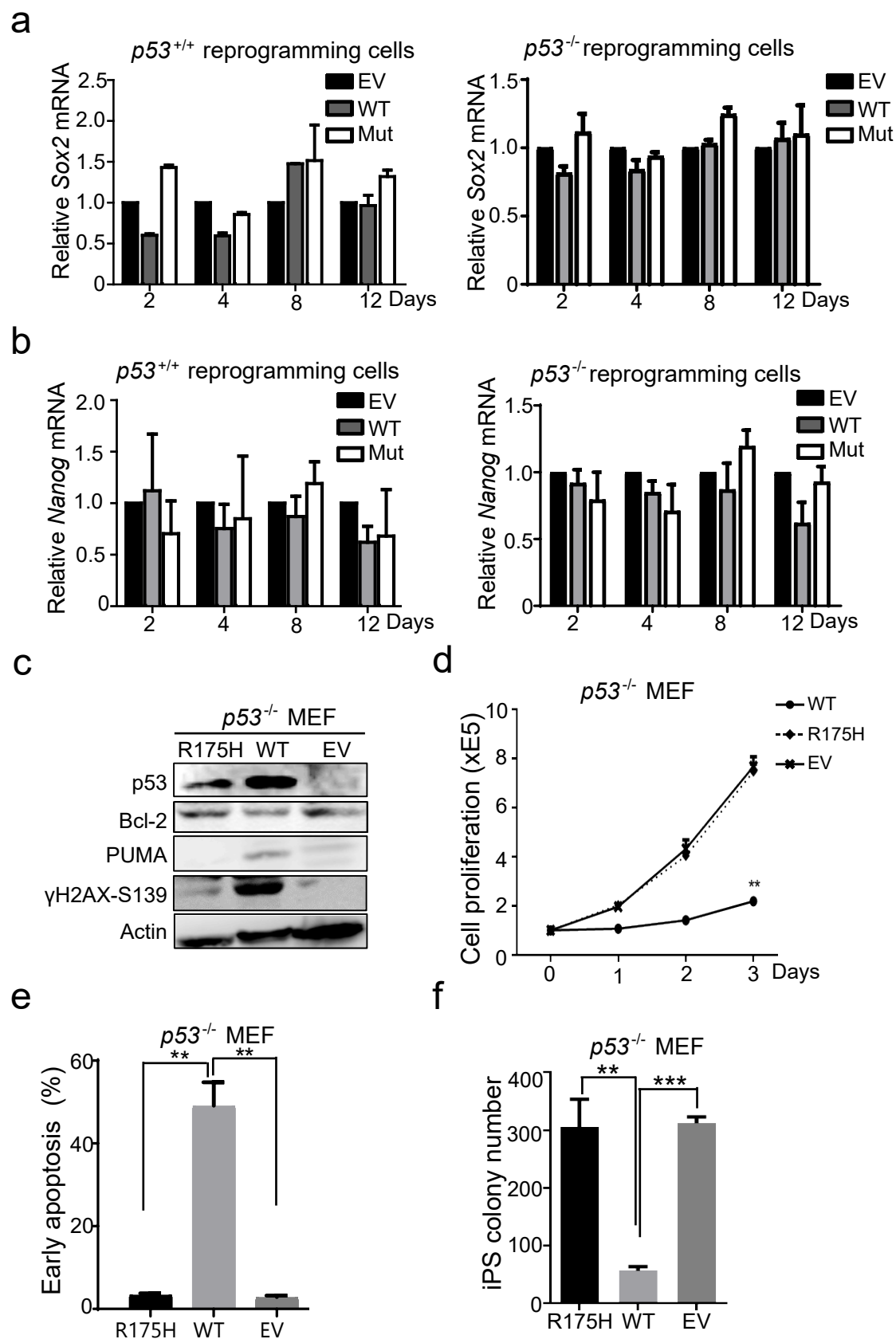

Fig. S1

Supplement: S1 Fig — a-b. RT-PCR of expression of SOX2 and NANOG in cells with p53 WT or mutant compared with an empty vector (EV) control. c. Western blot analysis of p53, BCL-2, and PUMA, γH2AX-139 expression after transfecting with lentiviruses carrying the p53 R175H, WT p53 and vector control plasmids into p53 KO MEF cells. d. Growth curve of p53 KO MEF cells with p53 WT or R175H. ** p<0.01. e. FACS analysis of apoptosis at day 3 after p53 KO MEF cells infection of p53 WT or R175H. ** p<0.01. f. iPS colony numbers following introduction of WT p53, R175H and vector into p53 KO MEFs were counted on reprogramming day 14 after transduction. (PDF) [file pone.0234262.s001.pdf]

Fig. S2

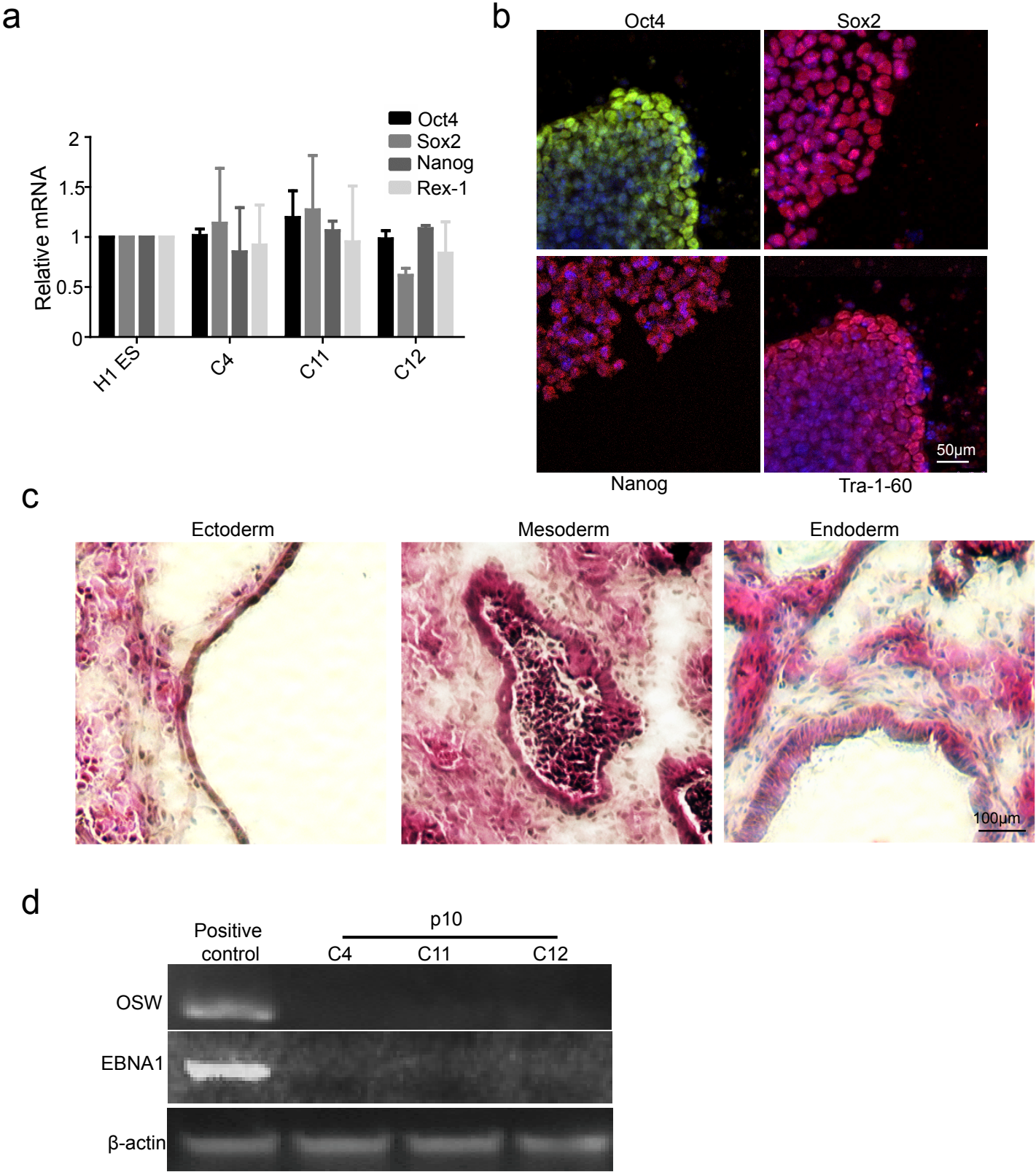

Supplement: S2 Fig — a. RT-PCR of expression of pluripotency genes in iPSCs compared with H1 ESCs. b. Representative images of pluripotency markers OCT4, SOX-2, NANOG, and TRA-1-60 in iPSCs. c. Teratoma analysis of iPSCs with p53 mutation. H&E staining of representative teratoma with derivatives of three embryonic germ layers: blood vessel with blood (mesoderm), glands (endoderm), and epithelium (ectoderm). d. Vector sequence (OSW and EBNA1) was tested by PCR-based detection in iPSCs expanded for 10 passages. (PDF) [file pone.0234262.s002.pdf]

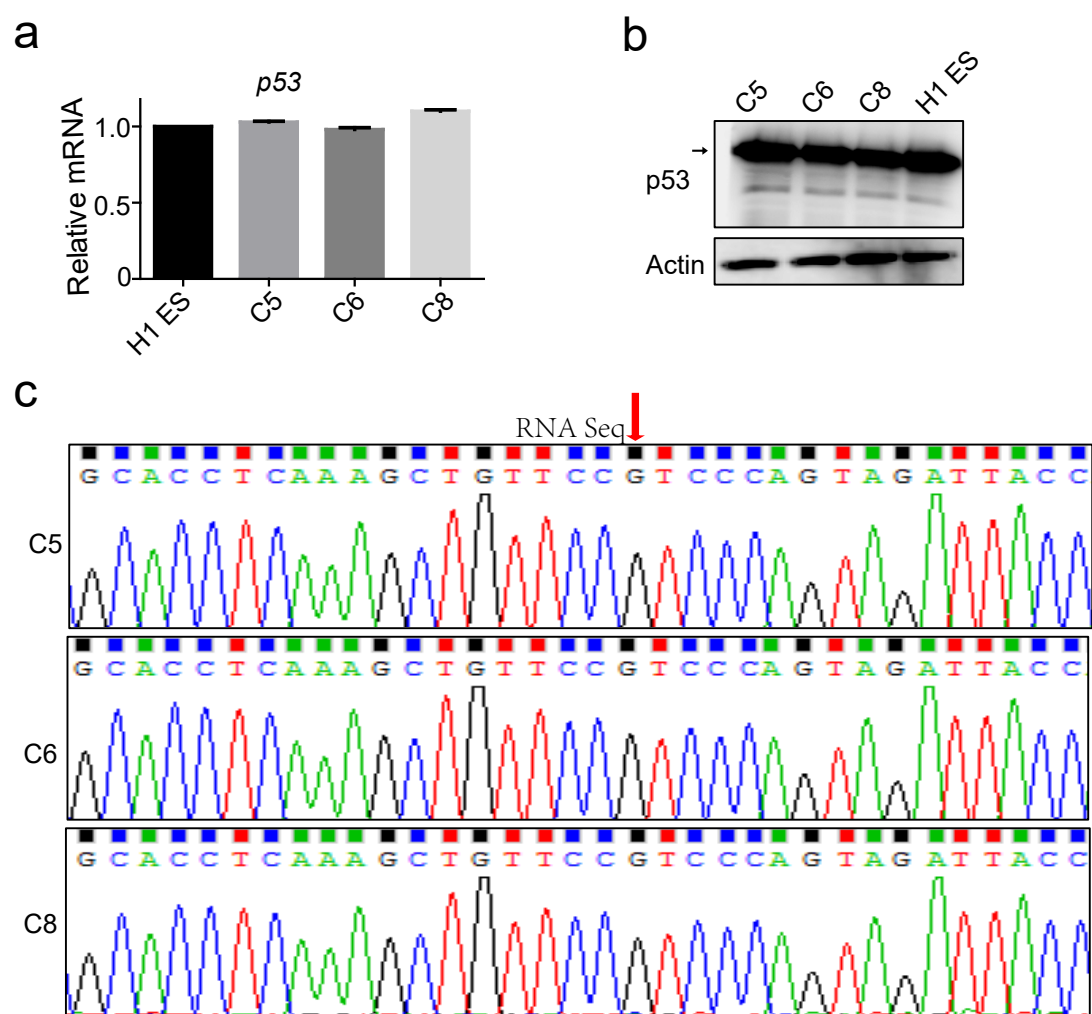

Fig. S3

Supplement: S3 Fig — a. RT-PCR of expression of p53 in another three iPS cell lines compared with H1 cells. b. WB of p53 protein levels in another three iPS cell lines compared with H1 cells. c. p53 cDNA sequence from another three iPS cell lines. (PDF) [file pone.0234262.s003.pdf]

Fig.2a

a

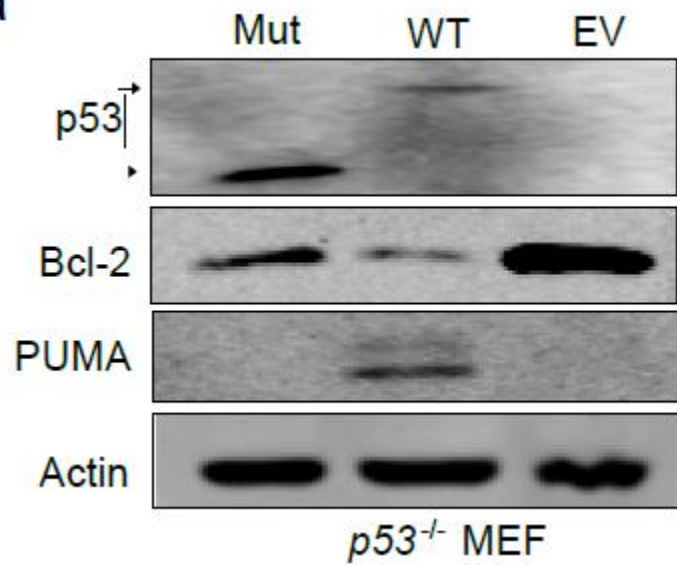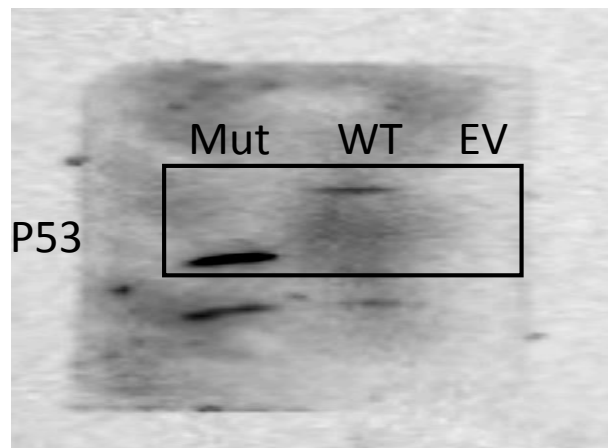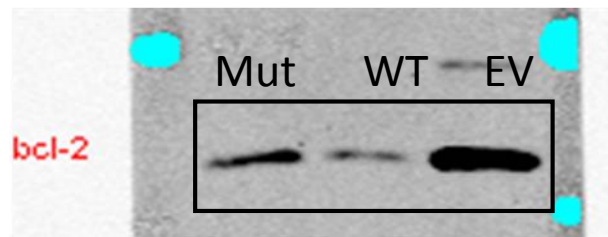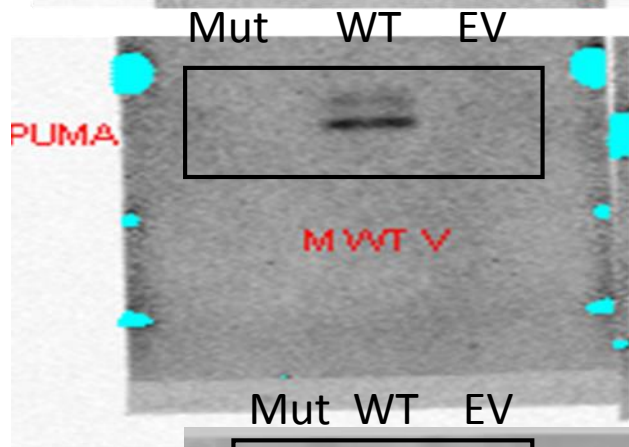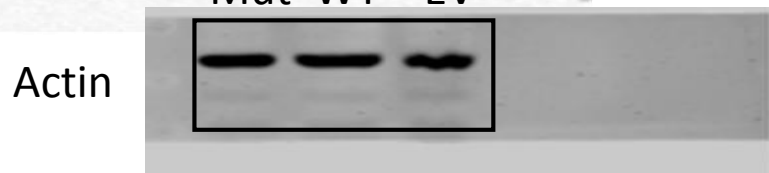

Fig.2d

d

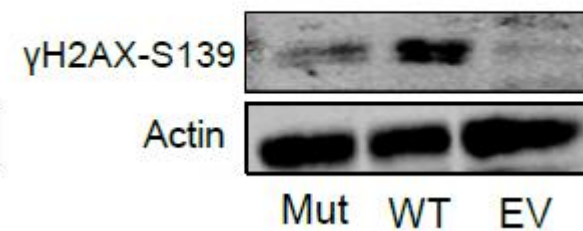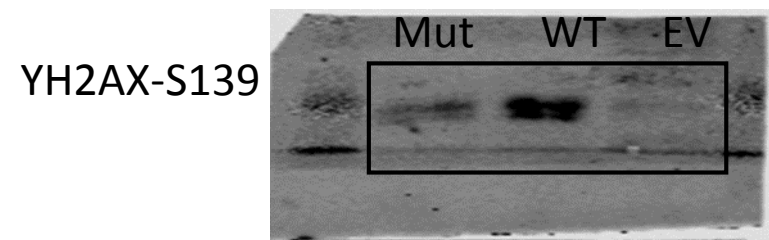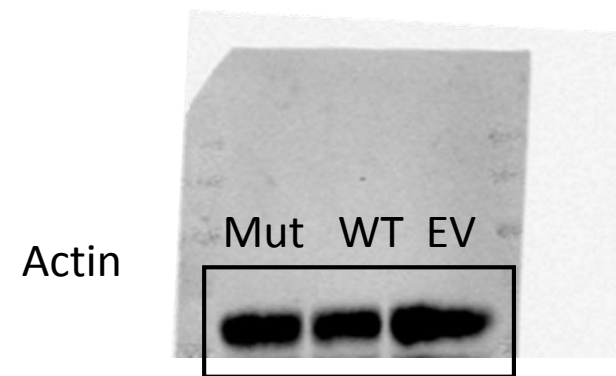

Fig.3

a

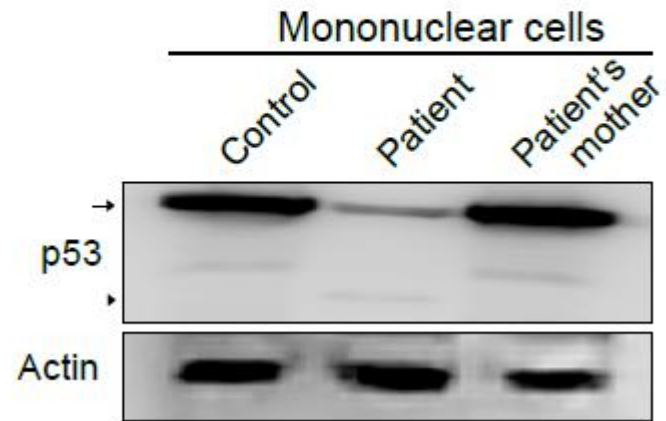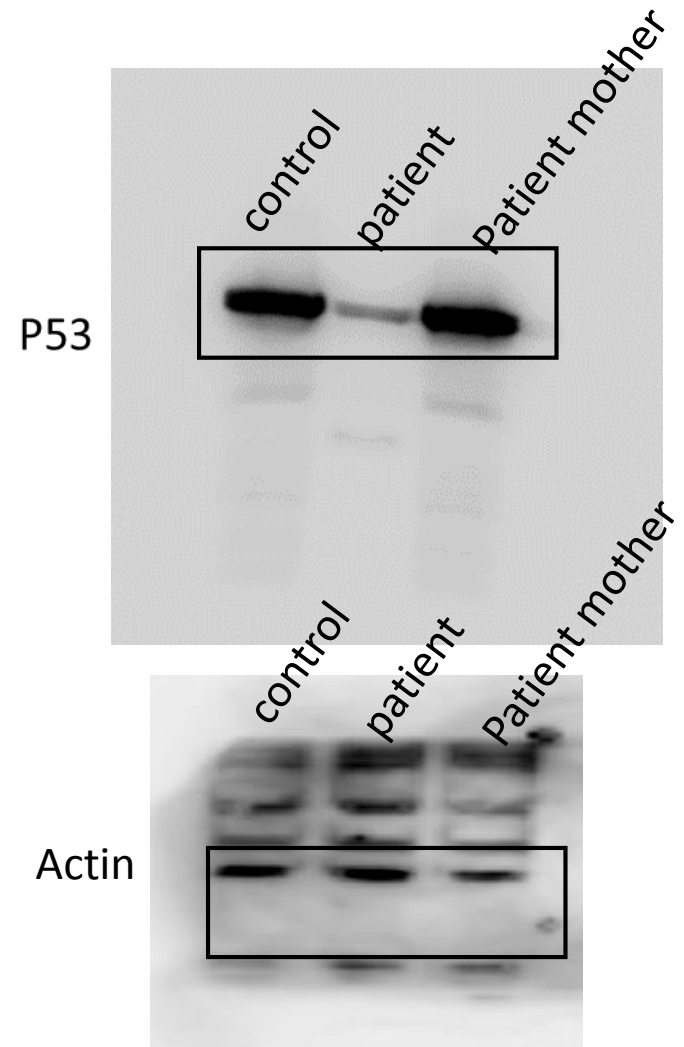

Fig.4C

C

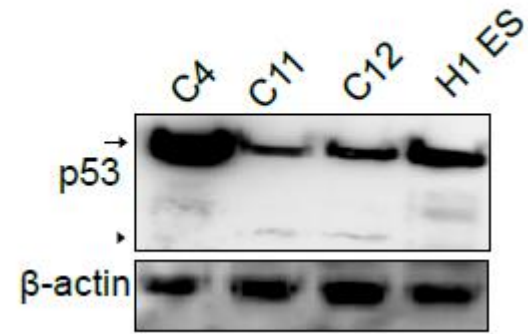

P53

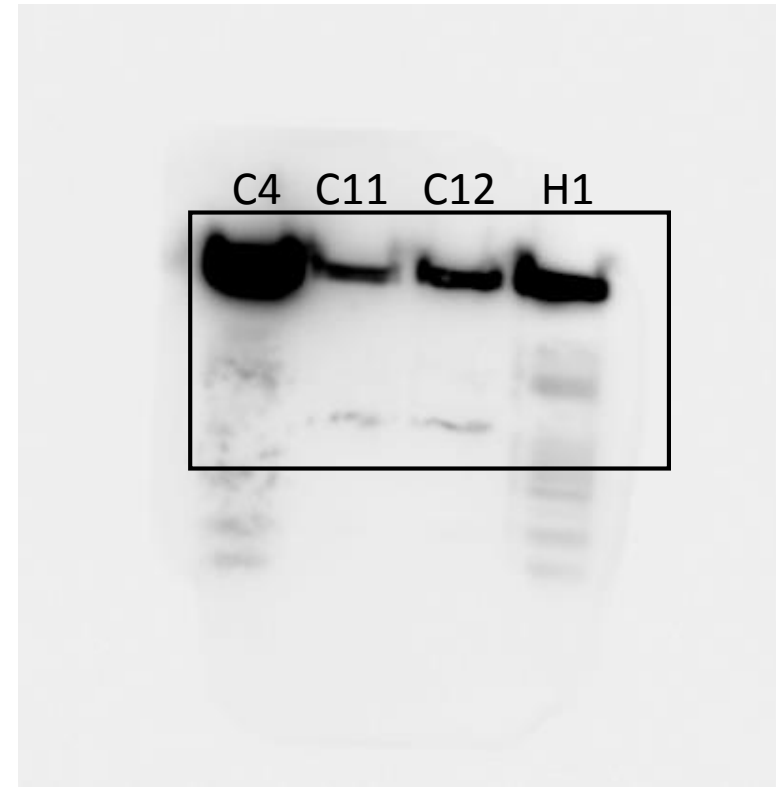

Actin

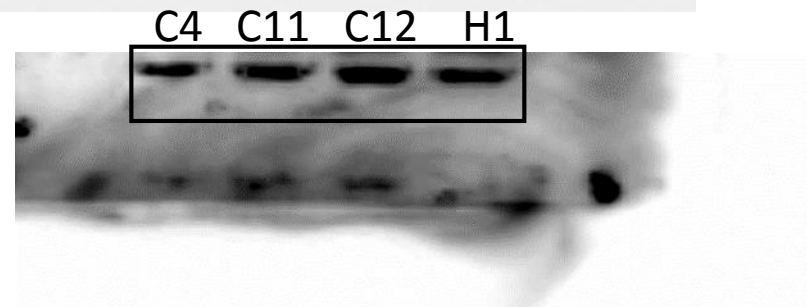

Supplemental Fig.1c

C

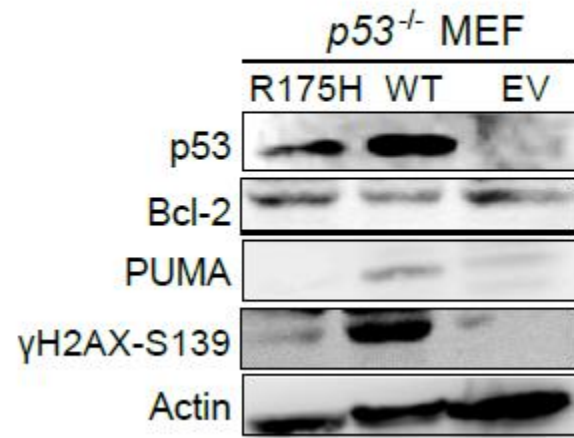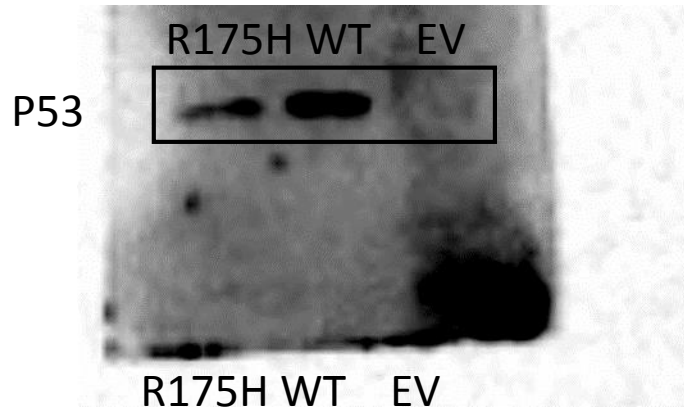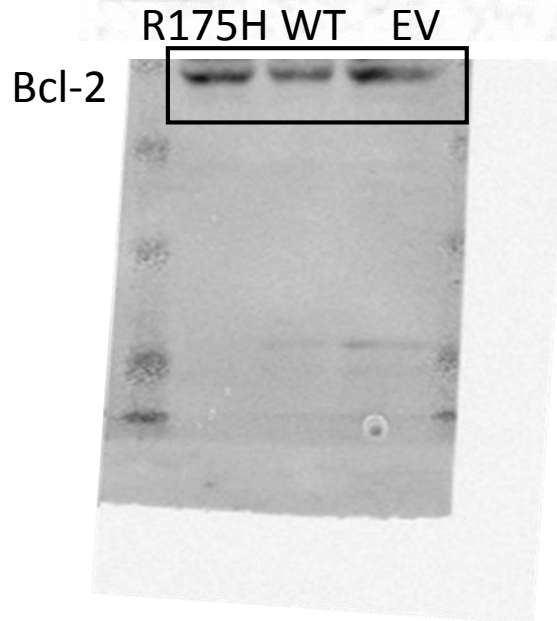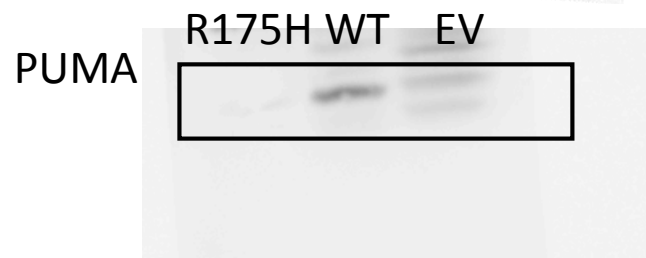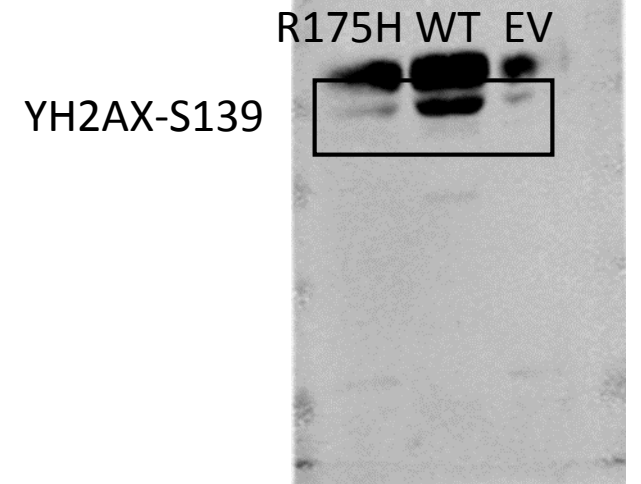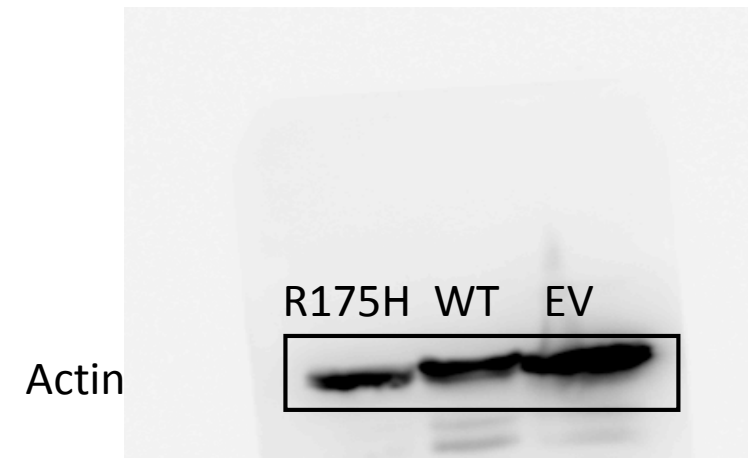

Supplemental Fig.3b

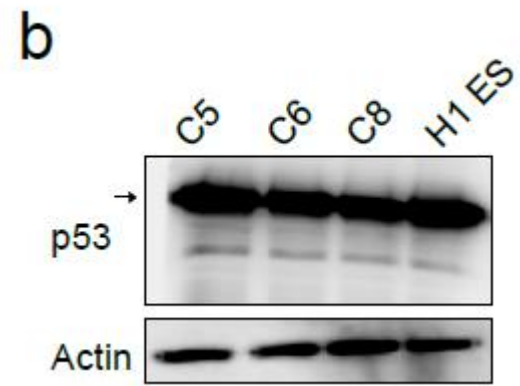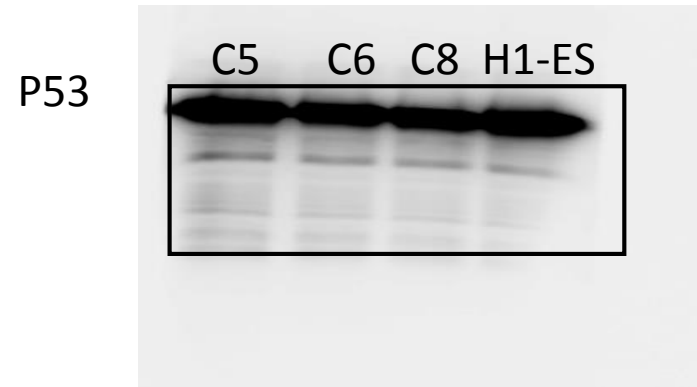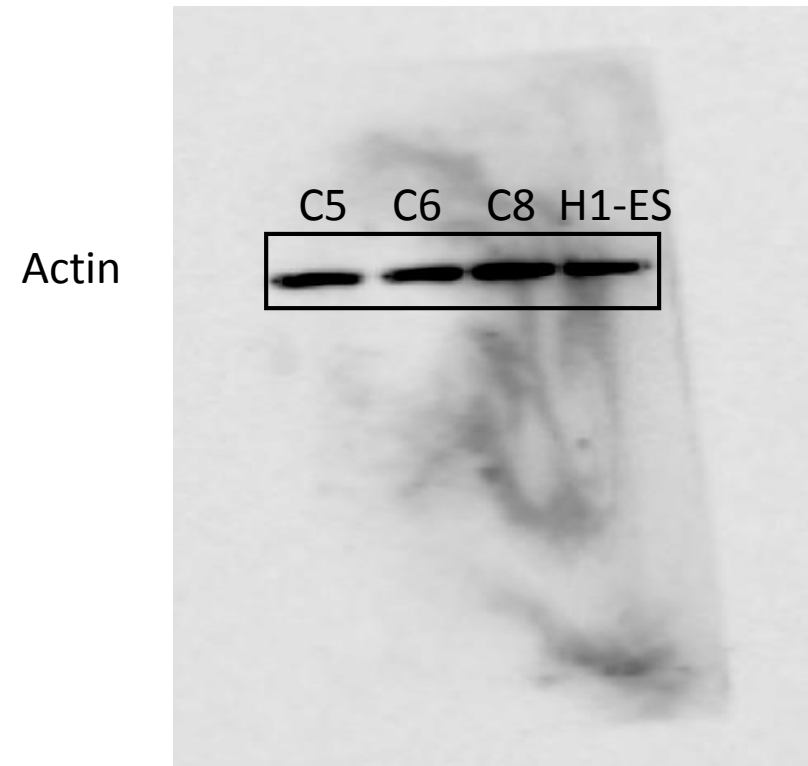

d

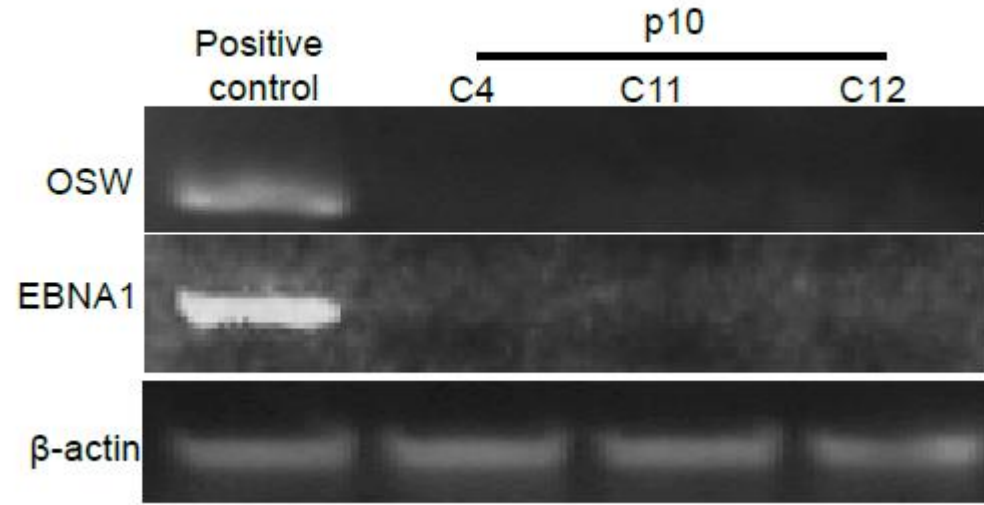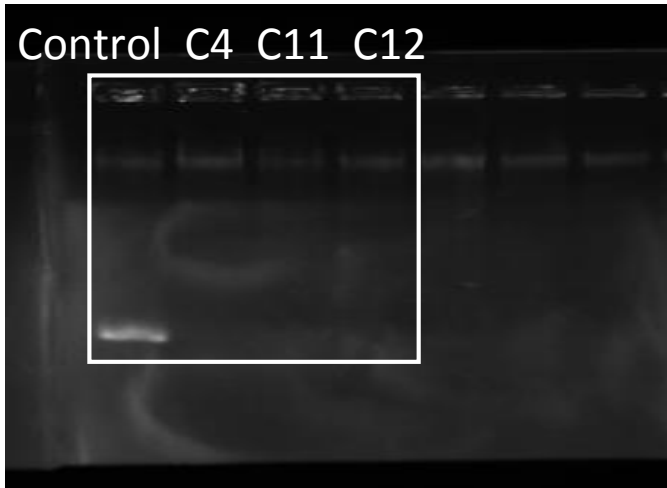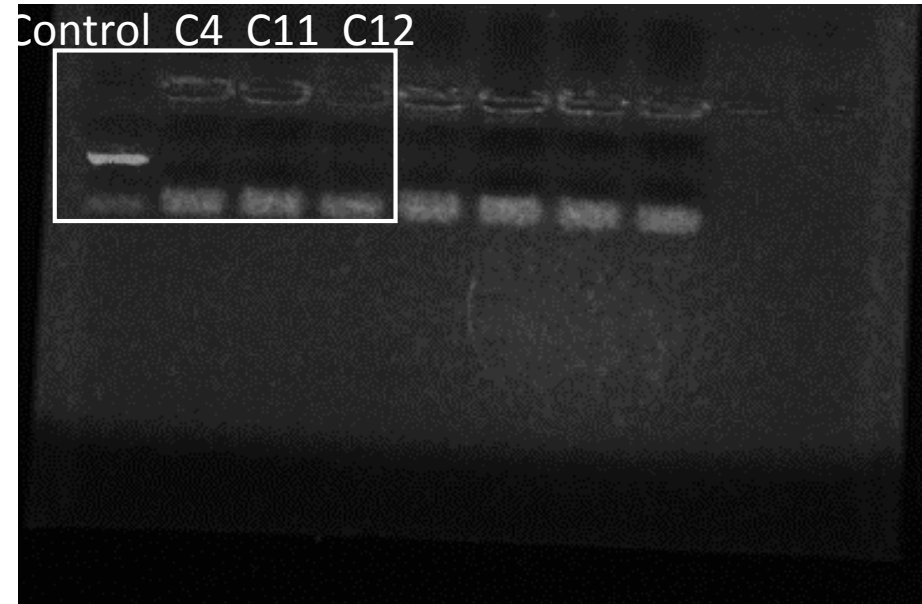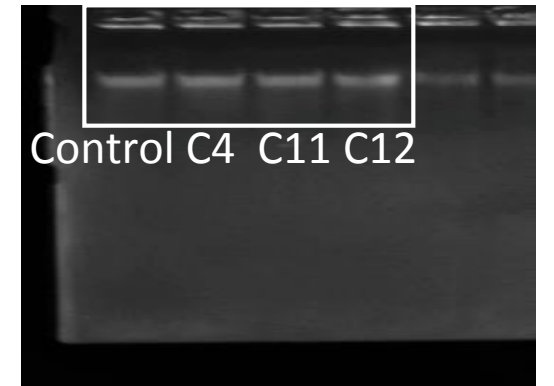

Supplement: S1 Raw Images — (PDF) [file pone.0234262.s004.pdf]
