# Supplementary material for: The function of a heterozygous p53 mutation in a Li-Fraumeni syndrome patient
Source: PLoS One. 2020 Jun 9;15(6):e0234262. doi: 10.1371/journal.pone.0234262 (PMC7282642; doi:10.1371/journal.pone.0234262)
Supplement: S1 Data — (DOCX) [file pone.0234262.s005.docx]

Fig.2b

| MEF-P53KO P53-MUT | | | MEF-P53KO P53-WT | | | MEF-P53KO P53-Vector | | |
| --- | --- | --- | --- | --- | --- | --- | --- | --- |
| 2.5 | 1.6 | 2.6 | 23.3 | 20.1 | 15.5 | 5.5 | 4.6 | 3.9 |

Fig.2c

|  | MEF-P53KO P53-WT | | | MEF-P53KO P53-MUT | | | MEF-P53KO control | | |
| --- | --- | --- | --- | --- | --- | --- | --- | --- | --- |
| 0 | 1 | 1 | 1 | 1 | 1 | 1 | 1 | 1 | 1 |
| 24 | 1.17 | 1.7 | 1 | 2.54 | 1.98 | 2.32 | 2.68 | 2.31 | 2.36 |
| 48 | 1.15 | 1.46 | 1.97 | 3.17 | 2.49 | 2.15 | 3.64 | 3.68 | 3.55 |
| 72 | 3.15 | 3.35 | 2.08 | 3.6 | 3.78 | 4.33 | 6.35 | 5.4 | 5.38 |

Fig.2e

| P53-MUT | P53-WT | P53-vector |
| --- | --- | --- |
| 0.222198 | 0.453357 | 0.193293 |
| 0.17952 | 0.4025 | 0.14354 |
| 0.19577 | 0.3121 | 0.1225 |

Fig.3b

| patient | patient's mom | Normal1 |
| --- | --- | --- |
| 10 | 30 | 46 |
| 9 | 29 | 65 |
| 10 | 30 | 68 |

Fig.3c

| patient | patient's mom | Normal1 |
| --- | --- | --- |
| 16 | 41 | 50 |
| 20 | 45 | 34 |
| 17 | 44 | 35 |

Fig.3d

| Ctr | C4 | C11 | C12 |
| --- | --- | --- | --- |
| 46 | 44 | 45 | 43 |
| 46 | 37 | 44 | 36 |
| 45 | 46 | 35 | 31 |
| 46 | 39 | 36 | 28 |
| 46 | 37 | 29 | 40 |
| 45 | 43 | 38 | 43 |
| 46 | 39 | 38 | 44 |
| 46 | 42 | 40 | 37 |
| 46 | 39 | 27 | 33 |
| 46 | 44 | 39 | 37 |
| 46 | 33 | 42 | 42 |
| 46 | 30 | 20 | 43 |
| 46 | 33 | 40 | 37 |
| 46 | 40 | 32 | 32 |
| 46 | 42 | 37 | 40 |
| 46 | 32 | 43 | 29 |
| 46 | 32 | 44 | 42 |
| 46 | 36 | 29 | 46 |
| 46 | 33 | 43 | 44 |
| 46 | 37 | 40 | 37 |

Fig.3e

|  | p53 WT MEF | |  | p53 KO MEF | |
| --- | --- | --- | --- | --- | --- |
| WT | Mutation | Vector | WT | Mutation | Vector |
| 20 | 25 | 54 | 48 | 50 | 300 |
| 26 | 23 | 50 | 57 | 55 | 315 |
| 23 | 19 | 51 | 63 | 62 | 321 |

Fig.3f

P53 WT

| days | Control | | WT | | M | |
| --- | --- | --- | --- | --- | --- | --- |
| 2 | 1 | 1 | 0.65 | 0.63 | 0.76 | 0.74 |
| 4 | 1 | 1 | 0.65 | 0.58 | 0.73 | 0.65 |
| 8 | 1 | 1 | 0.16 | 0.18 | 0.33 | 0.35 |
| 14 | 1 | 1 | 0.3 | 0.26 | 0.56 | 0.6 |

P53 KO

| days | Control | | WT | | M | |
| --- | --- | --- | --- | --- | --- | --- |
| 2 | 1 | 1 | 0.75 | 0.55 | 0.65 | 0.84 |
| 4 | 1 | 1 | 0.67 | 0.6 | 0.57 | 0.65 |
| 8 | 1 | 1 | 0.11 | 0.21 | 0.12 | 0.24 |
| 14 | 1 | 1 | 0.22 | 0.33 | 0.35 | 0.24 |

Fig.4b

| H9 | C4 | C11 | C12 |
| --- | --- | --- | --- |
| 1 | 1.63 | 1.38 | 1.76 |
| 1 | 1.24 | 1.96 | 1.27 |
| 1 | 1.27 | 1.4 | 1.38 |

Supplemental Fig. 1a

P53 WT

| days | Control | | WT | | M | |
| --- | --- | --- | --- | --- | --- | --- |
| 2 | 1 | 1 | 0.58 | 0.62 | 1.43 | 1.4 |
| 4 | 1 | 1 | 0.62 | 0.59 | 0.83 | 0.85 |
| 8 | 1 | 1 | 1.57 | 1.58 | 1.93 | 1.25 |
| 14 | 1 | 1 | 0.85 | 1.05 | 1.24 | 1.3 |

p53 KO

| day | Control | | M | | WT | |
| --- | --- | --- | --- | --- | --- | --- |
| 2 | 1 | 1 | 0.85 | 0.78 | 1.02 | 1.21 |
| 4 | 1 | 1 | 0.89 | 0.79 | 0.96 | 0.92 |
| 8 | 1 | 1 | 1.05 | 1.01 | 1.28 | 1.21 |
| 14 | 1 | 1 | 0.99 | 1.15 | 0.95 | 1.25 |

Supplemental Fig. 1b

P53 WT

| day | Control | | M | | WT | |
| --- | --- | --- | --- | --- | --- | --- |
| 2 | 1 | 1 | 0.73 | 1.51 | 0.48 | 0.93 |
| 4 | 1 | 1 | 0.92 | 0.59 | 0.42 | 1.28 |
| 8 | 1 | 1 | 0.73 | 1.01 | 1.34 | 1.04 |
| 14 | 1 | 1 | 0.73 | 0.51 | 0.36 | 1 |

P53 KO

| day | Control | | M | | WT | |
| --- | --- | --- | --- | --- | --- | --- |
| 2 | 1 | 1 | 0.85 | 0.99 | 0.65 | 0.94 |
| 4 | 1 | 1 | 0.91 | 0.79 | 0.57 | 0.85 |
| 8 | 1 | 1 | 0.73 | 1.01 | 1.28 | 1.11 |
| 14 | 1 | 1 | 0.73 | 0.51 | 0.85 | 1.01 |

Supplemental Fig. 1d

| time | P53-WT | | | P53-MUT | | | control | | |
| --- | --- | --- | --- | --- | --- | --- | --- | --- | --- |
| 0 | 1 | 1 | 1 | 1 | 1 | 1 | 1 | 1 | 1 |
| 24 | 1.05 | 0.83 | 1.32 | 1.91 | 2.2 | 1.98 | 1.9 | 1.98 | 2 |
| 48 | 1.18 | 1.67 | 1.39 | 4 | 3.73 | 4.36 | 3.86 | 4.05 | 5.03 |
| 72 | 2.15 | 2.37 | 2.04 | 7.98 | 7.06 | 7.36 | 7.57 | 7.04 | 8.4 |

Supplemental Fig. 1e

| M | | | W | | | Control | | |
| --- | --- | --- | --- | --- | --- | --- | --- | --- |
| 4.8 | 5.6 | 6.5 | 47.9 | 60.5 | 45.8 | 3.6 | 3.9 | 4.1 |

Supplemental Fig. 1f

| R175H | WT | Vector |
| --- | --- | --- |
| 218 | 46 | 287 |
| 311 | 52 | 302 |
| 385 | 72 | 341 |

Supplemental Fig. 2a

|  | Oct4 | | | Sox2 | | | Nanog | | | Rex-1 | | |
| --- | --- | --- | --- | --- | --- | --- | --- | --- | --- | --- | --- | --- |
| H1ESC | 1 | 1 | 1 | 1 | 1 | 1 | 1 | 1 | 1 | 1 | 1 | 1 |
| C4 | 0.99 | 1.09 | 0.97 | 1.34 | 0.52 | 1.56 | 0.88 | 1.28 | 0.4 | 1.31 | 0.94 | 0.51 |
| C11 | 0.9 | 1.41 | 1.28 | 1.63 | 0.65 | 1.54 | 1.15 | 0.96 | 1.08 | 1.31 | 1.24 | 0.31 |
| C12 | 0.99 | 1.06 | 0.9 | 0.54 | 0.63 | 0.68 | 1.06 | 1.12 | 1.07 | 0.66 | 0.66 | 1.2 |

Supplemental Fig. 3a

| H1 | C5 | C6 | C8 |
| --- | --- | --- | --- |
| 1 | 1.03 | 1.01 | 1.16 |
| 1 | 1.04 | 0.96 | 1.17 |
| 1 | 1.01 | 0.98 | 1.12 |
